# Effects of paternal high-fat diet and maternal rearing environment on the gut microbiota and behavior

**DOI:** 10.1038/s41598-022-14095-z

**Published:** 2022-06-17

**Authors:** Austin C. Korgan, Christine L. Foxx, Heraa Hashmi, Saydie A. Sago, Christopher E. Stamper, Jared D. Heinze, Elizabeth O’Leary, Jillian L. King, Tara S. Perrot, Christopher A. Lowry, Ian C. G. Weaver

**Affiliations:** 1 Department of Psychology and Neuroscience, Dalhousie University, Halifax, NS B3H 4R2 Canada; 2 Department of Integrative Physiology and Center for Microbial Exploration, University of Colorado Boulder, Boulder, CO 80309 USA; 3 Brain Repair Centre, Dalhousie University, Halifax, NS B3H 4R2 Canada; 4 Department of Psychology and Neuroscience and Center for Neuroscience, University of Colorado Boulder, Boulder, CO 80309 USA; 5 Department of Physical Medicine and Rehabilitation and Center for Neuroscience, University of Colorado Anschutz Medical Campus, Aurora, CO 80045 USA; 6 Veterans Health Administration, Rocky Mountain Mental Illness Research Education and Clinical Center (MIRECC), The Rocky Mountain Regional Veterans Affairs Medical Center (RMRVAMC), Aurora, CO 80045 USA; 7 Military and Veteran Microbiome Consortium for Research and Education (MVM-CoRE), Aurora, CO 80045 USA; 8 Department of Psychiatry, Dalhousie University, Halifax, NS B3H 4R2 Canada; 9 Department of Pathology, Dalhousie University, Halifax, NS B3H 4R2 Canada; 10 Present Address: The Jackson Laboratory, 600 Main Street, Bar Harbor, ME 04609 USA; 11 Oak Ridge Institute for Science and Education Research Participation Program, Oak Ridge, TN 37830 USA; 12 U.S. Department of Agriculture (USDA), National Animal Health Laboratory Network (NAHLN), Animal and Plant Health Inspection Service (APHIS), Ames, IA 50010 USA; 13 Present Address: Rocky Mountain MIRECC for Veteran Suicide Prevention, 1700 N Wheeling St, G-3-116M, Aurora, CO 80045 USA

**Keywords:** Stress signalling, Microbiome, Predictive markers, Rat, Feeding behaviour, Neuroscience, Epigenetics and behaviour

## Abstract

Exposing a male rat to an obesogenic high-fat diet (HFD) influences attractiveness to potential female mates, the subsequent interaction of female mates with infant offspring, and the development of stress-related behavioral and neural responses in offspring. To examine the stomach and fecal microbiome’s potential roles, fecal samples from 44 offspring and stomach samples from offspring and their fathers were collected and bacterial community composition was studied by 16 small subunit ribosomal RNA (16S rRNA) gene sequencing. Paternal diet (control, high-fat), maternal housing conditions (standard or semi-naturalistic housing), and maternal care (quality of nursing and other maternal behaviors) affected the within-subjects alpha-diversity of the offspring stomach and fecal microbiomes. We provide evidence from beta-diversity analyses that paternal diet and maternal behavior induced community-wide shifts to the adult offspring gut microbiome. Additionally, we show that paternal HFD significantly altered the adult offspring Firmicutes to Bacteroidetes ratio, an indicator of obesogenic potential in the gut microbiome. Additional machine-learning analyses indicated that microbial species driving these differences converged on *Bifidobacterium pseudolongum.* These results suggest that differences in early-life care induced by paternal diet and maternal care significantly influence the microbiota composition of offspring through the microbiota-gut-brain axis, having implications for adult stress reactivity.

## Introduction

As the obesity epidemic continues to intensify in Western societies, research has identified early life programming involving gene by environment (GxE) interactions underlying development of obesogenic phenotypes and susceptibility to high-fat diet (HFD)^1^. Western diets consist of large quantities of carbohydrates (from refined cereals, corn, potatoes and sugars, dairy products), fats and protein, and oils rich in omega-6 polyunsaturated fatty acids^2^. While the effects of HFD on individuals have been intensely studied over the past 30 years, the potential transgenerational impacts of HFD—more specifically, the impacts of paternal diet on obesogenic phenotypes in the offspring—are not well understood. Paternal HFD, leading to diet-induced obesity (DIO) in rats^3–5^, results in offspring with delayed growth, impaired liver function^6–10^, and deviations in social and anxiety-like defensive behavioral responses^11^, epigenetic reprogramming^12,13^, and alterations to the gut microbiome^14,15^. Recently, paternal diet has been implicated in the development of inter- and transgenerational offspring phenotypes^16–18^. While altered maternal investment^11,19–21^ could potentially explain offspring developmental deficits, epigenetic changes to spermatozoa that cause negative outcomes in the paternal lineage have been identified^22–29^. Additionally, probiotic treatment of male rodents in the paternal generation (F_0_) potentially buffers environmental stressors in F_1_ and F_2_ offspring. Combined, all of this evidence suggests that the paternal microbiome has a critical role in offspring development^30^.

GxE interactions influence the diversity of the gut microbiome^31–33^, thereby impacting HFD-induced metabolic phenotypes^34–37^. Mammalian development is dependent on GxE interactions within the context of infant-parent interactions, and environmental factors such as parental income, education, diet, exercise, and food availability influence the gut microbiome and neurodevelopment in humans^34,38–40^. GxE factors can be highly controlled in rodent models, allowing for investigation of how specific GxE mechanisms alter the microbiome and behavioral phenotypes^37,41,42^. Many studies have focused on how direct maternal influence impacts the developing microbiome and behaviors, and there is a need to better understand how paternal influence impacts offspring microbiome and behavior, as well any potential interactions between paternal quality and maternal investment^11,19,43^.

Mammalian maternal care has a profound influence on the early development of the offspring with life-long implications^44–47^. Both rodent and primate studies suggest that both the quantity and quality of infant-mother interactions are critical for early development^48–50^. The context (e.g., mate quality, environmental stress, previous maternal experience, rearing environment) of these infant-mother interactions can also influence the quantity and quality of care^11,43,51,52^. Recently, Bodden et al., found that paternal obesity decreased maternal care and was related to changes in offspring gut microbiota^15^. It is well accepted that neurodevelopment is susceptible to changes in early life, and the impacts of maternal care on neurodevelopment have been highly investigated^53–55^. More recently, it has been shown that the fetal and infant microbiome is also dynamic and shares similar susceptibilities to early life changes dependent on infant-mother interactions^5,56–58^. Maternal diet can also influence offspring microbiome diversity, as the ratio of Firmicutes to Bacteroidetes in HFD-fed mothers is shared by their non-HFD-fed offspring^59,60^. Clearly, the microbiota diversity of the mother is critical in the maintenance of the offspring microbiome, the developing gut-brain axis, and behavior^41,57,61,62^; but previous studies have not described the influence of maternal care or the rearing environment on these outcomes.

Prior work from our lab has demonstrated that paternal diet influences offspring development and physiological and behavioral responses to stress^11^. While we show that changes in stress reactivity may be linked to epigenetic modification of the *Crf* gene, other literature suggests that alterations to the microbiome influence stress responses^63–65^. Notably, mice raised in the absence of gut microbes have significant impairments in hypothalamic–pituitary–adrenal (HPA) axis regulation in response to restraint stress, including aberrant adrenocorticotropic hormone (ACTH) secretion. Further, when these mice are reconstituted with functional microbiota early in life, they show improved regulation of the HPA axis; which does not occur when reconstitution happens in adulthood^65^. Based on our previous research, early rearing environments and both paternal and maternal factors influence offspring stress reactivity^11,43^, and we hypothesize that this reactivity may be mediated by alterations to the offspring gut microbiome via both heritable and environmental mechanisms.

In the current study, we aimed to: (1) measure the impact of paternal HFD feeding on offspring gut microbiome diversity and community composition; (2) explore the impact and overlap of early-life rearing environment and maternal care on the offspring gut microbiome; and (3) identify potential interactions between paternal diet and postnatal rearing conditions on offspring gut microbiome and their response to predator odor-induced stress. As described previously^11^, we fed F_0_ males either control diet or HFD, bred them with females, and then measured maternal care of the offspring in the context of standard or semi-naturalistic housing (SNH) without the presence of the F_0_ male, and we determined that the diversity and community structure of the F_1_ offspring microbiome were significantly influenced by both paternal diet and maternal rearing environment. Together, these data support the hypothesis that paternal and maternal factors influence offspring gut microbiome and these changes influence behavior of the adult offspring.

## Methods

### Animals and breeding

All experimental procedures were performed in accordance with the guidelines of the Canadian Council on Animal Care and were approved by the Dalhousie University Committee on Laboratory Animals. Male (34) and female (30) Long-Evans hooded rats (64) (Charles River Canada, Sherbrooke, QC, Canada) were delivered to the vivarium at ~ 21–28 days old and maintained in the vivarium as same-sex pairs until adulthood (56 days old). All rats were maintained at 21 ± 2 °C under a 12 h reversed light cycle (lights off at 0930 h local time) in standard housing (SH), which consisted of polypropylene cages (47 cm length × 24 cm width × 20.5 cm height), wire lids, pine shavings for bedding (Hefler Forest Products, Inc., Sackville, NS, Canada), and a black polyvinyl chloride (PVC) tube (12 cm length × 9 cm diameter) for environmental enrichment, unless otherwise described (see below). For all rats, chow (Purina Lab Chow, Cat. No. 5001, Clarence Farm Services Ltd., Truro, NS, Canada) and water were supplied ad libitum. The research described here was conducted in compliance with the ARRIVE 2.0 Guidelines for Reporting Animal Research^66,67^. All efforts were made to limit the number of animals used and their suffering. Animals used in this study are identical to those used in a previous report; for a detailed description of animal procedures, see Korgan et al.^11^.

### Paternal high-fat diet

**Figure 1:**
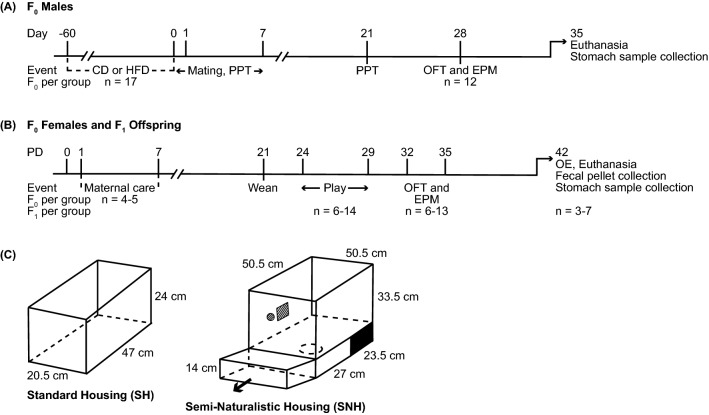
Experimental timelines and schematic representations of the housing conditions used for standard housing (SH) and semi-naturalistic housing (SNH). (**A**) Timeline of treatment procedures involving F_0_ males. Males were fed either control diet (CD) or high-fat diet (HFD) for 60 days prior to a partner preference test (PPT) using sexually receptive, naïve females. Within 12 h of the PPT, males were bred with different receptive naïve females. Following mating, males were removed and F_0_ females were left undisturbed until postnatal day (PD) 0. After mating, F_0_ males were maintained on standard chow for 21 days before a second PPT. One week after the second PPT, F_0_ male anxiety-like defensive behavioral responses were assessed in the open-field test (OFT) and the elevated plus-maze (EPM). Following behavioral testing, F_0_ males were immediately sacrificed; stomachs were collected, frozen and stored at − 80 °C for microbiome processing. (**B**) Timeline of treatment procedures for F_0_ females and the F_1_ offspring. After birth (postnatal day 0, PD0), offspring were counted, sexed, and weighed before being transferred to either fresh standard housing (SH) or semi-naturalistic housing (SNH), with biological mothers, until weaning. Maternal behavior (Maternal care) was scored for observational periods of 72 min, five times per day for seven consecutive days. At PD21, all offspring were weighed, weaned and placed in SH with a same-sex littermate. Play behavior was recorded in the home cage from PD24–29, followed by testing in the OFT and EPM on PD32 and 35, respectively. Predator odor exposure (OE) testing took place on PD42 with male and female offspring exposed to either a control odor (CO) or predator odor (PO) for 30 min, followed immediately by sacrifice. Fecal pellets and stomachs were collected, frozen and stored at − 80 °C for microbiome processing. Sample sizes are provided in the figure for both F_0_ and F_1_ groups. (**C**) Schematics of the SH and SNH. The SNH consisted of a lower burrow compartment contained within a drawer that could be moved out to facilitate cage cleaning and an upper section that contained food and water; the two sections were connected by a hole (visible in the upper section). *CD* control diet, *CO* control odor, *EPM* elevated plus-maze, *HFD* high-fat diet, *OE* odor exposure, *OFT* open-field test, *PD* postnatal day, *PO* predator odor, *PPT* partner preference test, *SH* standard housing, *SNH* semi-naturalistic housing. Adapted from Korgan et al.^11^, with permission (License Number 5047130532142).

A timeline of the experimental procedures is shown in Fig. 1A,B. F_0_ males began either high-fat (60 kcal %; Product No. D12492) or protein/carbohydrate-matched control diet (10 kcal%; Product No. D12450J) feeding on PD35 and remained on this diet for 60 days (Research Diets, Inc., New Brunswick, NJ, USA). This diet is commonly used to induce DIO in rodents and mirrors the increased dietary fat and sugar content of a western diet^11,68,69^. Paired F_0_ male cage-mates were randomly assigned either control diet (CD, *n* = 17) or high-fat diet (HFD, *n* = 17). On day 0, all F_0_ males were supplied with standard rodent chow (Purina Lab Chow, Cat. No. 5001, Clarence Farm Services Ltd., Truro, NS, Canada) and water ad libitum until sacrifice.

### F_0_ breeding

For breeding (Fig. 1A), one male and one naïve female (~ 56 days old), determined to be in estrus, were housed together for seven consecutive days; the male was then removed from the maternal cage. The day of birth was defined as postnatal day (PD) 0; pups remained with the dam until weaning at PD21 (Fig. 1B), upon which the F_1_ offspring were re-housed in same-sex littermate pairs.

### Semi-naturalistic housing (SNH)

F_0_ females mated with CD and HFD F_0_ males were observed daily for pups beginning at gestational day 20 (GD20), near the beginning of the dark cycle^11^. Once F_1_ offspring arrived (PD0), the litter was counted, sexed, and weighed as quickly as possible to minimize disruption. F_0_ females and F_1_ offspring randomly designated for the SNH condition (*n* = 9 dams, *n* = 40 offspring) were transferred to SNH cages on PD0. Dams and pups in the SH condition (*n* = 10 dams, *n* = 38 offspring) were placed in clean SH (Fig. 1C). All F_0_ dams and F_1_ pups were left undisturbed until the pups were weaned at PD21. The SNH (Fig. 1C) consisted of two sections: an upper section (50.5 cm width × 50.5 cm length × 33.5 cm height) containing chow food and water ad libitum and a lower section (50.5 cm width × 50.5 cm length × 14 cm height) filled with pine shavings and a polyvinyl chloride (PVC) tube.

### Maternal care observations

Maternal behaviors of F_0_ females were scored daily in real-time for 72 min at 0800 h, 1100 h, 1300 h, 1500 h, and 2130 h from PD1 to PD7 (Fig. 1B). During each observation period, the frequencies of the behaviors were determined using an instantaneous sampling method, recording the behavior occurring every 3 min^11,47^. For the current study, we assessed arched-back-nursing (ABN), with licking and grooming (LG) of pups. ABN consisted of graded degrees of arching, levels 2–4, based on the presence of kyphosis (bending of the knees and back arching) while nursing F_1_ offspring. The sum of high-quality maternal behaviors towards the offspring (i.e., the total number of occurrences of LG, ABN2, and ABN3; representing moderate and moderate/high degrees of kyphosis) were divided into three groups to form equal sized “high”, “medium”, and “low” maternal care groups for further analyses.

### Offspring groups

The following four groups of F_1_ male and F_1_ female offspring were utilized: (1) CD-SH—F_0_ male exposed to control diet and F_0_ female housed in standard housing (*n* = 14 males, 11 females); (2) CD-SNH—F_0_ male exposed to control diet and F_0_ female housed in semi-naturalistic housing (*n* = 8 males, *n* = 10 females); (3) HFD-SH—F_0_ male exposed to high-fat diet and F_0_ female housed in standard housing (*n* = 6 males, *n* = 7 females); and (4) HFD-SNH—F_0_ male exposed to high-fat diet and F_0_ female housed in semi-naturalistic housing (*n* = 10 males, *n* = 12 females). Groups of F_1_ offspring contained *n* = 2–4 (per sex) from multiple litters: CD-SH (*n* = 6 litters); CD-SNH (*n* = 4 litters); HFD-SH (*n* = 4 litters); and HFD-SNH (*n* = 5 litters).

### Acute stress exposure in peri-adolescent offspring and fecal microbiome sample collection

We used the Predator Odor Exposure Test to identify ethologically relevant differences in responses to acute stress exposure^43,70^. Testing of responses to acute stress exposure in F_1_ offspring in the odor exposure (OE) arena (clear Plexiglas walls and lid with ventilation holes and white Plexiglas floor; 60 cm × 27 cm × 35.5 cm) was conducted on PD42. F_1_ offspring were randomly assigned to either predator odor (PO; cat urine) or control odor (CO) exposure (*n* = 3–7 per sex, per group). Offspring were exposed for one 30-min trial. Fecal samples from each F_1_ offspring rat were removed directly from the OE arena and immediately frozen at − 80 °C until further processing. At harvest, whole stomachs were dissected and flash-frozen on dry-ice and shipped to the University of Colorado Boulder. Between each trial, the OE arena was cleaned with 30% ethanol solution.

### Stomach microbiome sample collection

Stomach microbiome sample collection was conducted in a sterile BSL2 cabinet using sterile technique. Stomachs, individually wrapped in aluminum foil, were removed from the − 80 °C freezer, thawed on wet ice, and then dissected along the lesser curvature with a sterile scalpel blade (Cat. No. 320001, Fisher Scientific LLC, Denver, CO, USA). To minimize risk of inter-sample contamination, scalpel blades were discarded immediately after use into a sharps container to prevent each blade from being used more than once. The incision along the lesser curvature was held open with sterile, disposable forceps while a BD BBL™ CultureSwab™ EZ sterile polyurethane single swab (Cat No. 220144, Becton, Dickinson and Company, Franklin Lakes, NJ, USA) was used to swab the gastric mucosal surface and contents of the stomach. The swabs were immediately placed in individual sterile tubes (Cat. No. 76332–058 2 ml tubes, VWR), labeled, and frozen at − 80 °C until molecular processing for analysis of the stomach microbiome.

### Molecular processing of microbiome samples

DNA from stomach and fecal samples was extracted using a PowerSoil DNA extraction kit (Cat. Nos. 12888-100 and 12955-4, MoBio Laboratories, Carlsbad, CA, USA) according to manufacturer’s instructions. Marker genes in isolated DNA were PCR-amplified using GoTaq^®^ Master Mix (Cat. No. M7133, Promega, Madison, WI, USA) and 515-bp forward (5ʹ-GTGCCAGCMGCCGCGGTAA-3ʹ)/806-bp reverse (5ʹ-GGACTACHVGGGTWTCTAAT-3ʹ) primer pair (Integrated DNA Technologies, Coralville, IA, USA) targeting the V4 hypervariable region of the 16 small subunit ribosomal RNA (16S rRNA) gene, modified with a unique 12-base sequence identifier for each sample and the Illumina adapter, as previously described by Caporaso et al.^71^. The thermal cycling program consisted of an initial step at 94 °C for 3 min, followed by 35 cycles (94 °C for 45 s, 55 °C for 1 min, and 72 °C for 1.5 min) and a final extension at 72 °C for 10 min. PCR reactions were run in duplicate; products from the duplicate reactions were pooled and visualized on an agarose gel to ensure successful amplification. PCR products were cleaned and normalized using a SequalPrep Normalization Kit (Cat. No. A1051001, ThermoFisher, Waltham, MA, USA) following manufacturer’s instructions. The normalized amplicon pool was sequenced on an Illumina MiSeq, run using V3 chemistry, 600 cycles, and 2 × 250-bp paired-end sequencing. All sequencing and quality control assessments were conducted at the University of Colorado Boulder BioFrontiers Institute Next-Gen Sequencing Core Facility (Boulder, CO, USA).

### Stomach and fecal microbiome analysis

Demultiplexed, quality-filtered reads were analyzed using the QIIME 2 2019.4 workflow with default parameters^72^, generating error-corrected amplicon sequence variants (ASVs) using the DADA2 (ver. 1.10.0) denoising and dereplication workflow^73^.

Stomach samples averaged 6758.7 ± 436.8 (mean ± standard error of the mean) sequences per sample, with the minimum number of sequences required to retain a sample in the study set at 2200 for F_0_ sires and 1850 reads for F_1_ offspring to maximize the number of samples retained. Except where noted, the raw OTU table was rarefied to correct for differential sequencing effort and resultant library size artifacts. Although rarefaction is a conservative approach that limits power for discovery of differences, it more clearly clusters samples according to biological origin in presence/absence ordinations (i.e., unweighted UniFrac) than other normalization techniques and is an essential prerequisite to conducting α- and β-diversity analyses^74^. Seven samples were excluded due to insufficient sequence reads (less than 90) from the following animal subjects: Rat IDs 9, 18, 19, 21, 26, 28, and 36. Reads from the 34 remaining samples were clustered into sub-OTUs against the Greengenes 13/8 reference database as above. Representative sequences were then aligned with MAFFT and a phylogenetic tree was constructed with FastTree 2 for phylogenetic diversity calculations. In summary, microbial data from 34 of the original 41 stomach samples were analyzed for a variety of alpha diversity metrics (for details, see below).

Fecal samples averaged 9381.3 ± 619.7 (mean ± standard error of the mean) sequences per sample, with the minimum number of sequences required to retain a sample in the study set at 3136 reads to maximize the number of samples retained. Four samples were excluded due to insufficient sequence reads (less than 2000) from the following animal subjects: Rat IDs 43, 59, 67, and 77. Reads from the 44 remaining fecal samples were clustered into sub-OTUs against the Greengenes 13/8 reference database^75,76^. Representative sequences were then aligned with MAFFT (ver.7.0)^77^, and a phylogenetic tree was constructed with FastTree 2 (ver. 2.0)^78^ for phylogenetic diversity calculations. In summary, microbial data from 44 of the original 48 fecal samples were analyzed for a variety of alpha diversity and beta diversity metrics (for details, see below). Additionally, we utilized a supervised machine-learning algorithm to predictively classify individual fecal microbiome samples of male and female offspring as belonging to either F_0_ paternal CD or HFD, F_0_ maternal/ early-life F_1_ offspring housing SH or SNH, or F_0_ maternal care quality; high, medium, or low. Using a nested cross-validated (k = fivefold) strategy, the receiver-operating characteristic (ROC) area-under-the-curve (AUC) values for the ASV-based models that were used to calculate feature importances varied between 0.77 and 0.95.

### Statistical analyses

Statistical analysis in QIIME2 of each alpha-diversity metric made available in the command-line interface was conducted on both the stomach and fecal microbiome sequencing data (Chao1 richness estimator, Shannon’s entropy *Hʹ*, Menhinick’s richness index, and Simpson’s evenness index.

Briefly, once each alpha-diversity metric above was calculated for stomach microbiome samples from F_0_ sires and F_1_ offspring and fecal samples from F_1_ offspring, statistical analysis was conducted using Kruskal–Wallis tests and post hoc pairwise Mann–Whitney U tests with adjustment for multiple comparisons on all categorical variables in the metadata. For the stomach sample metadata from the paternal generation/F_0_ sires, these variables included: final weight at necropsy, dietary condition (CD or HFD), weight gain from pre- to post-diet manipulation, testes weight, abdominal fat pad weight, gonadal fat weight, brain weight, and percentages of each weight taken relative to the final weight at necropsy.

For both the stomach and fecal sample metadata relevant to only the F_1_ offspring, we conducted statistical analysis as described above against the following variables: sex (male or female), filial generation (F_0_ or F_1_), paternal dietary condition (CD or HFD), F_0_ maternal/early-life F_1_ offspring housing (SH or SNH), F_1_ stress exposure condition (CO or PO), F_0_ maternal care quality (licking/grooming and arched-back nursing), *Crf* hnRNA expression, and expression of H3K9ac at the *Crf* promoter. In the fecal sample metadata, F_1_ offspring-specific behavioral outcomes in the elevated plus-maze (EPM) and the open-field (OF) test were also collected and analyzed. These included: time spent in the EPM open arms (in s and as a % of total EPM time), line crosses in the EPM, entry frequency into the EPM open arms, grooming frequency in the EPM, rearing frequency in the EPM, line crosses in the OF, time spent in the center of the OF (in s and as a% of total OF time), grooming frequency in the OF, and rearing frequency in the OF. F_0_ sire and F_1_ offspring behavioral metadata for the EPM and OF as just described were also included in the alpha-diversity analysis of the stomach microbiome sequencing data. Only the significant drivers of variation in the F_1_ offspring are reported in the results section and can be found in Supplementary Tables 1–3. Alpha-diversity analyses of *Crf* hnRNA expression and H3K9ac at the *Crf* promoter, and behavioral metadata for the EPM and OF were found to be not significant. Raw data points for each of the metadata columns described can be found in Supplementary File 2 and 3.

Additional statistical analysis of core and commonly reported alpha-diversity metrics (number of distinct features, Shannon’s entropy, and Faith’s phylogenetic diversity) using 3-way linear mixed effects models and post hoc pairwise *t*-tests without adjustment for multiple testing were also conducted on the fecal microbiome sequencing data. Following rarefaction of the ASV table generated by 16S rRNA gene sequencing of all fecal samples briefly described above, we conducted a linear mixed effects model-based 3-way analysis of F_0_ paternal diet (CD or HFD), F_0_ maternal/early-life F_1_ offspring housing (SH or SNH), and F_1_ stress exposure conditions (CO or PO). For beta diversity distances, such as weighted and unweighted UniFrac, similarly structured 3-way permutational analyses of variance of F_0_ paternal diet, F_0_ maternal/early-life F_1_ offspring housing, and F_1_ stress exposure conditions were conducted using the R package *adonis* (999 Monte Carlo permutations).

## Results

### Paternal HFD influences offspring weight and anxiety-like behavior

**Figure 2:**
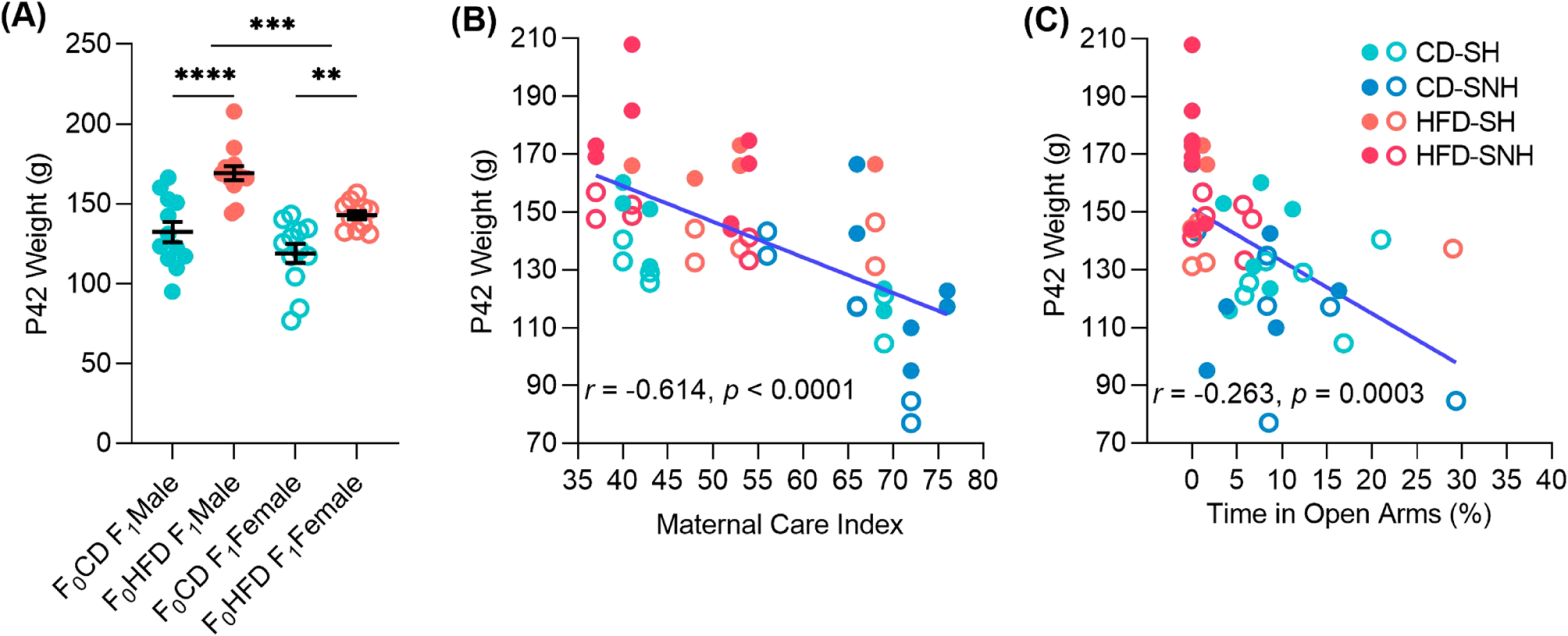
F_1_ offspring weight is associated with paternal dietary treatment, maternal behaviors, and anxiety-like behaviors in the EPM. (**A**) F_1_ offspring weights are dependent on F0 paternal dietary treatment with HFD. Data shown represent means + standard errors for each group. (**B**) Pearson’s correlation of F_0_ maternal behaviors (scored for observational periods of 72 min, 5 times per day for 7 days from PD1 to 7 as a maternal care index of licking, grooming, and arched-back nursing) with adult F_1_ offspring weights taken at the time of sacrifice. Points represent data collected from individual rats (blue = F_1_ offspring of control diet (CD) sires, orange = F_1_ offspring of high-fat diet (HFD) sires). (**C**) Pearson’s correlation of time spent in open arms of the elevated plus maze (EPM) against adult F_1_ offspring weights taken at the time of sacrifice. Points represent data collected from individual rats (blue = F_1_ offspring of control diet (CD) sires, orange = F_1_ offspring of high-fat diet (HFD) sires). Statistical significance was evaluated using a pairwise t-test within F_1_ sexes. **p < 0.01, ****p < 0.0001. *CD* control diet, *HFD* high-fat diet.

We showed that male and female F_1_ offspring of F_0_ HFD sires were significantly heavier on PD42 compared to offspring of F_0_ CD sires (one-way analysis of variance, *F*_(3, 44)_ = 17.91, *p* < 0.0001; Fig. 2A). We also showed that F_1_ male offspring weighed more than F_1_ female offspring (two-way analysis of variance, *F*_(1,44)_, *p* = 0.0004) and that male and female F_1_ offspring of F_0_ HFD sires were significantly heavier on PD42 compared to male and female offspring, respectively, of F_0_ CD sires (two-way analysis of variance, *F*_(1, 44)_ = 17.91, *p* < 0.0001; Fig. 2A); this is despite the fact that F_1_ offspring were fed control chow from weaning through PD42. These data are consistent with previous findings indicating that F_0_ paternal HFD feeding negatively impacted mating success and subsequent F_0_ maternal care of F_1_ offspring^11,43^ which has also been shown to affect F_1_ offspring bodyweight^51^. Additionally, we examined whether correlations exist between maternal care, F_0_ paternal HFD treatment, offspring anxiety-related defensive behavioral responses, and F_1_ offspring weight (PD42). Pearson’s correlation analysis demonstrated that increased frequency of F_0_ maternal behaviors scored pre-weaning (PD1–7) were inversely associated with F_1_ offspring weights taken at PD42 (*r* =  − 0.614, *p* < 0.0001; Fig. 2B). Further, body weight was negatively correlated with percentage of time in the open arms of the elevated plus-maze, (*r* =  − 0.263*, p* = 0.0003; Fig. 2C). Together, these results highlight the link between F_0_ maternal care, F_0_ paternal diet, and anxiety-like behavior in F_1_ offspring.

### Maternal housing and anxiety-like behaviors in offspring impact the gastric microbiota

Although the stomach microbiome is known to have lower diversity than the intestinal microbiome, we selected the stomach microbiome as representative of the microbial continuity of the aerodigestive tract^79^, which is thought to play an important, albeit understudied, role in modulation of physiology and behavior. We conducted Kruskal–Wallis tests and post hoc pairwise Mann–Whitney U tests with adjustment for multiple testing on all categorical variables in the metadata describing each sample submitted for 16S rRNA gene sequencing of the stomach microbiomes. Samples were collected from F_0_ CD and HFD sires and F_1_ offspring from F_0_ paternal diet (CD or HFD), F_0_ maternal/ early-life F_1_ offspring housing (SH or SNH), and F_1_ stress exposure conditions (CO or PO). A full summary of significant findings for F_0_ CD and HFD sires is in Supplemental Table 1; significant findings for F_1_ offspring are summarized in Supplemental Table 2.

Briefly, the most consistent effects that we observed among F_0_ sires were differences in multiple alpha-diversity metrics of evenness and richness in association with diet (Shannon’s *H*ʹ, *p* = 0.028, *H* = 4.80) and rearing behaviors in the open-field arena (Shannon’s *H*ʹ, *p* = 0.028, *H* = 4.80).

Differences in multiple alpha-diversity measures of evenness and richness were also observed to be associated with early-life environmental and behavioral test outcomes in the F_1_ offspring population. These included: F_0_ maternal/F_1_ early-life housing conditions (Chao1, *p* = 0.027, *H* = 4.86 and Simpson’s evenness, *p* = 0.007, *H* = 7.34), rearing in the open-field arena (Chao1, *p* = 0.014, *H* = 6.06 and Menhinick, *p* = 0.023, *H* = 5.14), and rearing in the elevated plus-maze (Simpson’s evenness, *p* = 0.041, *H* = 4.18). Interestingly, differences in alpha-diversity due to sex (*p* = 0.023, *H* = 5.14) and stress response in the odor exposure test (*p* = 0.023, *H* = 5.14) were observed in Simpson’s evenness for F_1_ offspring.

### Paternal diet, maternal housing, and stress exposure interactions impact gut microbial phylogenetic diversity and richness

Because previous literature indicates that changes in stress responsivity and body weight may be associated with changes in the gut microbiome, we conducted three-way linear mixed-effects models to evaluate the potential main and interactive effects of F_0_ paternal diet (CD or HFD), F_0_ maternal/early-life F_1_ offspring housing (SH or SNH), and F_1_ stress exposure conditions (CO or PO) on microbial alpha-diversity. As described in “Molecular processing of microbiome samples” section, the 16S rRNA gene V4 amplicon data were first subsampled at a rarefaction depth of 3136 reads per fecal sample collected from 44 F_1_ offspring in the test arena at the conclusion of the odor exposure (OE) test, immediately prior to sacrifice.

**Figure 3:**
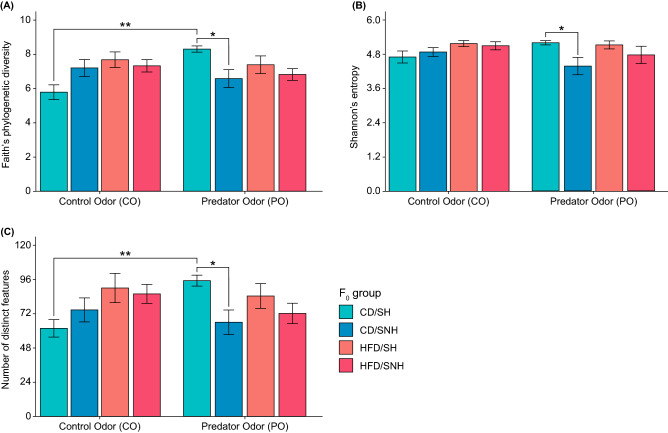
Within-subjects fecal microbiome analyses of alpha-diversity among F_1_ offspring are dependent on paternal F_0_ conditions. Analyses of alpha-diversity as measures of microbial richness include. (**A**) Faith’s phylogenetic diversity, (**B**) Shannon’s entropy, and (**C**) number of distinct features. Bars represent means ± standard errors of the means for each group. Three-way linear mixed-effects models evaluating individual and interaction effects of paternal diet, maternal housing, and stress exposure conditions were performed on 16 small subunit ribosomal RNA (16S rRNA) gene V4 amplicon data at a rarefaction depth of 3,136 reads per fecal sample collected from individual F_1_ offspring in the test arena at the conclusion of the predator odor exposure (OE) test, just before sacrifice. *CD* control diet, *HFD* high-fat diet, *OE* odor exposure, *SH* standard housing, *SNH* semi-naturalistic housing. **p < 0.01, ***p < 0.001, pairwise Wilcoxon rank-sums tests without multiple corrections.

Analysis of Faith’s phylogenetic diversity revealed interaction effects of paternal diet × maternal housing × stress exposure (*F*_(1,36)_ = 6.2, *p* = 0.018; Fig. 3A), paternal diet × stress exposure (*F*_(1,36)_ = 5.3, *p* = 0.016; Fig. 3A), and maternal housing × stress exposure (*F*_(1,36)_ = 6.4, *p* = 0.027; Fig. 3A). Analysis of Shannon’s entropy revealed the presence of significant interaction effects of maternal housing × stress exposure (*F*_(1,36)_ = 4.2, *p* = 0.049; Fig. 3B), while analysis of the number of distinct features revealed a paternal diet × stress exposure interaction (*F*_(1, 36)_ = 4.9, *p* = 0.034; Fig. 3C). However, no main effects of F_0_ paternal diet, F_0_ maternal/ early-life F_1_ offspring housing, or F_1_ stress exposure were found in any of the linear mixed-effects models described above.

### Paternal diet with maternal housing or stress exposure impacts global composition of the gut microbiota

**Figure 4:**
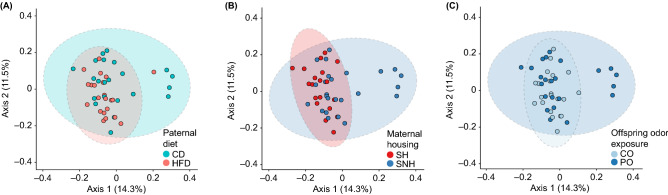
Emperor plots of unweighted unique fraction metric (UniFrac) distances and principal coordinates analyses (PCoA) demonstrate that between-subjects beta diversities differ among F_1_ offspring depending on paternal F_0_ conditions, maternal housing conditions, and stress exposure. PCoA axes 1 and 2 explain 25.8% of the variation shown here among samples grouped by (**A**) paternal diet (blue = control diet, CD; orange = high-fat diet, HFD), (**B**) maternal housing conditions (red = standard housing, SH; dark blue = semi-naturalistic housing, SNH), and (**C**) stress in the predator odor exposure (OE) test on postnatal day 42 (PD42) (light blue = control odor, CO; dark blue = predator odor, PO). Each sample is represented as a single point in principal coordinate space; points that are closer together are more similar than those that are further apart. Dashed ellipses represent 95% confidence intervals around the centroid mean and are filled with the color corresponding to each group. *OE* odor exposure, *PCoA* principal coordinates analyses, *PD* postnatal day, *SH* standard housing, *SNH* semi-naturalistic housing.

To assess effects of paternal diet, maternal housing conditions, and stress exposure on the community composition of fecal microbiomes, we conducted three-way permutational analysis of variance (999 permutations) using the R package *adonis* on the unweighted and weighted UniFrac distance matrices generated from the rarefied feature table. Analysis of weighted UniFrac in the between-subjects comparisons showed no significant differences in microbial beta-diversity (data not shown). However, we demonstrated the presence of an interaction effect of paternal diet × maternal housing condition (*F*_(1,36)_ = 1.59, *r*^2^ = 0.036, *p* = 0.044; Fig. 4A,B) and paternal diet × stress exposure (*F*_(1,36)_ = 1.52, *r*^2^ = 0.034, *p* = 0.050; Fig. 4A,C) in the unweighted UniFrac distance matrix data.

### HFD offspring have elevated Firmicutes to Bacteroidetes ratios compared to CD offspring

**Figure 5:**
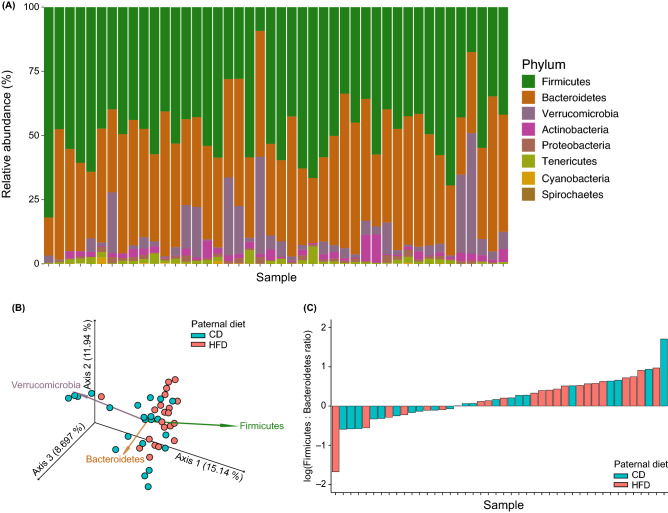
Ratios of relative abundances of the two most dominant phyla, Firmicutes and Bacteroidetes, differ between F_1_ offspring of sires fed control diet (CD) and F_1_ offspring of sires fed high-fat diet (HFD). (**A**) Stacked bar plot showing the relative abundances (%) of phyla observed in each sample, represented as individual columns. (**B**) Compositional biplot vectors show that Firmicutes and Bacteroidetes, in particular, drive differences in F_1_ offspring fecal microbiomes. Biplot vectors are superimposed over the unweighted UniFrac distances shown in this Emperor plot after a rarefaction depth of 3136 sequences was applied to the data. (**C**) Samples ordered by increasing log values of the Firmicutes: Bacteroidetes ratio and colored according to paternal diet condition (CD or HFD) demonstrate that F_1_ offspring of HFD sires have greater Firmicutes to Bacteroidetes (log10F:B ratio) > 0 than F_1_ offspring of CD sires. *CD* control diet, *HFD* high-fat diet.

The relative abundances of all phyla identified in the rarefied feature table of offspring fecal microbiomes are shown in Fig. 5A. From the stacked barplot, over 90% of the gut microbiota of all 44 F_1_ offspring fecal samples were members of the phyla Firmicutes, Bacteroidetes, and Verrucomicrobia. We generated a principal coordinates analysis (PCoA) plot of the unweighted unique fraction metric (UniFrac) distances with compositional biplot vectors representing the contributions of these 3 phyla to beta-diversity clustering patterns in the offspring of CD and HFD sires (Figs. 4A, 5B). Here, we demonstrated that high levels of Firmicutes corresponded to fecal samples belonging to F_1_ offspring of HFD sires (Fig. 5B). Analyses of the fecal samples of F_1_ offspring with log-transformed relative abundance ratios of Firmicutes to Bacteroidetes (log_10_F:B ratio) > 0 demonstrated that 16 samples belonged to progeny of F_0_ HFD sires and an additional 11 samples with (log_10_F:B ratios) > 0 belonging to progeny of F_0_ CD sires had greater than 10% prevalence of Verrucomicrobia (Fig. 5A,C). Fisher’s Exact probability test identified a significant difference between the relative abundance ratios of Firmicutes to Bacteroidetes (log_10_F:B ratio) > 0 for CD and HFD F_1_ offspring (OR 4.49, *p* = 0.033), with a greater number of F_0_ HFD sires having Firmicutes to Bacteroidetes (log_10_F:B ratio) > 0.

### Using microbial features in the offspring fecal microbiota to accurately predict parental conditions

**Figure 6:**
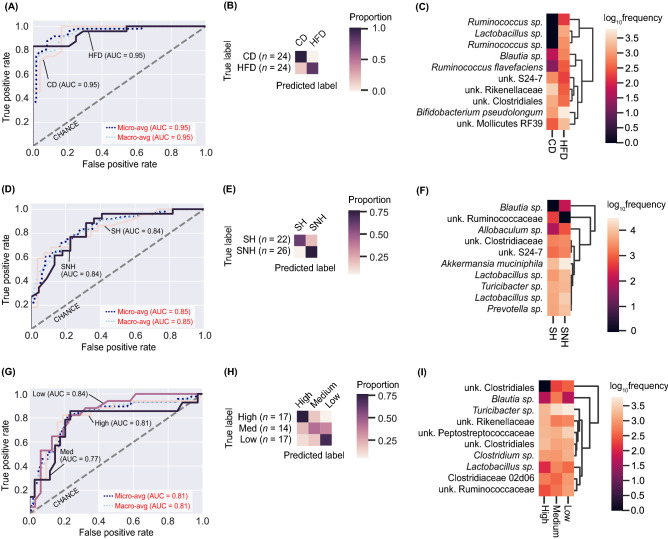
Machine learning reliably predicts (**A–C**) F_0_ paternal dietary condition (control diet/CD or high-fat diet/HFD), (**D–F**) F_0_ maternal housing condition (standard housing/SH or semi-naturalistic housing/SNH), and (**G–I**) maternal care levels (high, medium, or low) based on differential features in the F_1_ offspring gut microbiome. Maternal care categories are based on a composite score of licking, grooming, and arched-back nursing scored during observational periods of 72 min, 5 times a day, for 7 consecutive days from postnatal days PD1–7. Feature inputs from the raw feature table included in this analysis consisted of all bacteria matched to the Greengenes 13/8 ribosomal ribonucleic acid (RNA) reference database with 99% accuracy. (**A,D,G**) Per-class receiver operating characteristic (ROC) plots demonstrate micro-average precision (averaged metrics across each sample, dark blue dashed line), macro-average precision (equal weights assigned to each classification level, light blue dashed line), and classification error rate achieved by random chance (grey dashed line) at several true-positive rate (TPR) against false-positive rate (FPR) thresholds. Predictive precision results for each classification level are shown in nude ((**A**), control diet, CD; (**D**), standard housing, SH; (**G**), high maternal care, High), dark purple ((**A**), high-fat diet, HFD; (**D**), semi-naturalistic housing, SNH; (**G**), moderate maternal care, Medium), and light purple ((**G**), low maternal care, Low); overall classification accuracy was greater than 80% in each model. (**B,E,H**) Prediction matrices show which samples were correctly classified (true label and predicted labels match) and which samples were incorrectly classified (true label and predicted labels mismatch) by the model; darker hues correspond to more accurate assessments of the true group designation for all samples. (**C,F,I**) Feature heatmaps show microbial features that were used to classify samples in each category after all parameters were tuned and optimized for the greatest predictive accuracy. Heatmap colors indicate log_10_frequency of each feature in the dataset. *AUC* area-under-the-curve, *CD* control diet, *FPR* false-positive rate, *HFD* high-fat diet, *PD* postnatal day, *ROC* receiver operating characteristics, *TPR* true-positive rate.

A nested cross-validation random forest approach (5 k-mers) was used to evaluate sample classification by F_0_ condition (paternal diet, Fig. 6A–C; maternal housing condition, Fig. 6D–F; maternal care index, Fig. 6G–I). Unbiased classification of samples based on microbial composition extended our understanding of how the fecal microbiota of F_1_ rat offspring in this study were influenced by F_0_ conditions beyond core diversity analyses and relied on predictive accuracy scores and machine learning model performance indicators in the receiver operating characteristic (ROC) scores (Fig. 6A,D,G).

Briefly, macro- and micro-average values for the prediction of samples to F_1_ offspring of CD or HFD sires based on features of the fecal microbiome were greater than chance alone (AUC = 0.95 and 0.95, respectively; Fig. 6A) and 95% accurate (Fig. 6B). The feature importance plot in Fig. 6C shows that the presence or absence of *Ruminococcus* and *Lactobacillus* spp. together drove the classification of samples into paternal CD or HFD progeny. Additional features included *Blautia* sp., *Ruminococcus flavefaciens, Bifidobacterium pseudolongum*, and unknown members of the Bacteroidetes class S24-7, family *Rikenellaceae*, order *Clostridiales,* and order *Mollicutes* RF39 (Fig. 6C).

Macro- and micro-average values for the prediction of samples to F_1_ offspring reared with dams in SH or SNH environments based on features of the fecal microbiome were also greater than chance alone (AUC = 0.85 and 0.85, respectively; Fig. 6D) and 84% accurate (Fig. 6E). The feature importance plot in Fig. 6F demonstrates that there exists some overlap in features used in the classification of samples by paternal diet and maternal housing condition, including *Blautia* sp. and an unknown member of the Bacteroidetes class S24-7 (Fig. 6C,F). Unique features that drove the classification of SH and SNH conditions included unknown members of the family Ruminococcaceae and Clostridiaceae*,* *Allobaculum* sp., *Turicibacter* sp., *Prevotella* sp., *Akkermansia muciniphila*, and multiple features in the genus *Lactobacillus* (Fig. 6F).

Macro- and micro-average values for the prediction of samples to F_1_ offspring observed to have been reared in high, medium, or low maternal care conditions based on features of the fecal microbiome were additionally greater than chance alone (AUC = 0.81, 0.77, and 0.84, respectively; Fig. 6G). Predictive accuracies for each classification level shown in Fig. 6G and the confusion matrix in Fig. 6H demonstrate that the medium maternal care group was the most difficult assignment based on microbial features alone (AUC = 0.77). However, accuracy scores for the high and low maternal care groups were greatly improved (AUC = 0.81 and 0.84, respectively; Fig. 6G). The feature importance plot in Fig. 6I demonstrates that here, too, there is substantial overlap in features used in the classification of samples by maternal care and housing condition, including *Blautia* sp., *Turicibacter* sp., and an unknown member of the family Ruminococcaceae. As in prior analyses, there were also some unique features that drove the classification of samples into recipients of high, medium, or low maternal care such as *Lactobacillus* sp. and *Clostridium* sp. (Fig. 6I). Additional unique features that were not well characterized at the species level included unknown members of the order Clostridiales, family Peptostreptococcaceae, and sub-family Clostridiaceae 02d06 (Fig. 6I).

**Figure 7:**
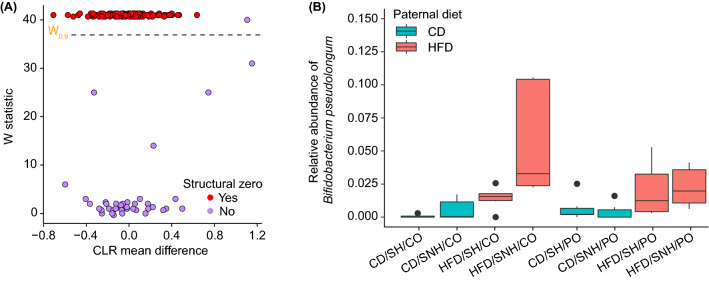
*Bifidobacterium pseudolongum* significantly contributes to differential microbiome compositions observed among F_1_ offspring of control diet (CD) and high-fat diet (HFD) F_0_ sires. (**A**) Analysis of composition of microbiomes (ANCOM-II) volcano plot showing the mean center-log ratio (CLR) differences in abundance and W-statistics for taxa that are differentially abundant in a test of F_0_ paternal dietary condition × maternal housing condition. Significant taxa that drive differences in the model lie above the coefficient of concordance threshold of 0.9 (horizontal black dashed-line; purple circle). Note that some significant taxa are also defined as structural zeros (red circles; Mandal et al.^80^). Non-significant taxa lie under the threshold. (**B**) Relative abundance plot of *Bifidobacterium pseudolongum*, the only significant taxon detected in the ANCOM-II analysis. The horizontal black lines indicate the medians; bottoms and tops of boxes indicate the first and third quartiles, respectively; whiskers indicate the 1.5 interquartile range beyond the upper and lower quartiles. Values outside the whiskers are indicated by black circles. *ANCOM-II* analysis of composition of microbiomes, *CD* control diet, *CLR* center-log ratio, *CO* control odor, *HFD* high-fat diet, *PO* predator odor, *SH* standard housing, *SNH* semi-naturalistic housing.

Linear mixed-effects model analysis of paternal diet and maternal housing condition using ANCOM-II identified one significant feature above the coefficient of concordance threshold of 0.9 in the F_1_ fecal microbiome data (Fig. 7A). The observed relative abundance of *Bifidobacterium pseudolongum* (*W*-statistic = 40) in the offspring of HFD sires was significantly higher compared to offspring of CD sires in all subgroups irrespective of maternal housing condition or odor exposure prior to sacrifice (Fig. 7B).

## Discussion

In the current study, we have identified novel interactions between preconception paternal diet, postnatal rearing conditions, maternal care, and predator odor-induced stress that impact the offspring microbiome. Specifically, we have identified correlations between paternal diet factors and maternal investment which influence offspring weight and anxiety-like behavior, microbiome alpha- and beta-diversity, and Firmicutes to Bacteroidetes ratios. Further, we identified that compositional dynamics between members of the phyla Firmicutes and Bacteroidetes principally drove these treatment group differences, while also identifying *Bifidobacterium pseudolongum*, a member of the phylum Actinobacteria, as an important individual species that was differentially-abundant based on paternal diet condition.

Correlational analyses suggest that there was an interaction between paternal diet and maternal investment, and these interactions influence offspring weight throughout life and adult anxiety-like behavior. Maternal investment is dictated by many factors, including the quality of the mate and previous and current environmental conditions^20,21,81^. Previously, we identified decreases in the quality of maternal care in offspring from stressed or HFD-fed F_0_ males and that deviations in maternal investments have long-term impacts on offspring growth and behavior^11,43^. Conversely, offspring of males in an enrichened environment received higher quality maternal care and had improved learning and memory^20,82^. We identified significant interactions in F_1_ male and female offspring weight; predictably, (1) males weighed more than females, (2) offspring of F_0_ sires fed HFD weighed more than offspring of F_0_ sires fed CD. This weight increase was negatively correlated with the quality of maternal care and associated with increased anxiety-like behavior; these findings suggest there is an interaction between paternal diet, early-life experience, maternal investment, and adult anxiety-like behavior. While previous studies have suggested that high quality environment can influence maternal care and offspring development^51,83^, acute stress exposure in peri-adolescent rats, relative to unstressed controls, has not been extensively studied to identify differences in behavior, gene expression, epigenetic regulation, and gut microbiota composition. To probe the potential interactions of paternal diet, maternal care/offspring weaning environment, and acute stress exposure on the gut microbiome, we analyzed fecal samples from F_1_ offspring following predator odor exposure (or control).

Next, we demonstrated that there are interactive effects of paternal diet and maternal investment on offspring gut microbial alpha-diversity. Previous studies of paternal and maternal HFD feeding have detailed robust differences in alpha-diversity in F_1_ offspring^60,84^. While we do not replicate the diet-induced differences in diversity, our model excluded maternal HFD-feeding and eliminated HFD metabolites in breast milk, unlike other studies. Other studies with controlled paternal-only feeding have shown similarly limited effects of paternal prebiotic^85^ and sucrose diets^86^ on alpha diversity in F_1_ offspring microbiomes. Interestingly, we identified several significant alterations in alpha diversity based on offspring odor exposure. The acute nature (30 min) of the PO exposure, immediately prior to sample collections, suggests the possibility of stress-induced catecholaminergic effects on microbiome diversity^87,88^ that are dependent on offspring rearing environment.

We found that these interaction effects were also observed in compositional analyses of microbial beta-diversity, such that paternal high-fat diet drives the presence and absence of keystone species in offspring. Again, like previous studies of paternal and/or maternal HFD feeding^84,89^, we show a more subtle effect of paternal HFD on offspring gut microbiomes, similar to paternal prebiotic^85^ and sucrose diets^86^. We identified a profound influence of rearing environment, supporting previous evidence that identified early life care and stress as key drivers of offspring microbiome beta-diversity^57,90^. We also identified beta diversity differences based on PO exposure, again suggesting an impact of catecholaminergic signaling on acute microbiome changes^87,88^.

We demonstrated that paternal HFD exposure increases the ratio of Firmicutes to Bacteroidetes in offspring, agreeing with previous epigenetic studies^60^. This subtle shift in the prevalence of Firmicutes also reflects the effects seen in obesity and prenatal stress^90,91^ and further supports the role of paternal nutrition in shaping offspring growth and behavior^7,10,11,22,25,85,92,93^. The role of other predominant phyla in the rat gut microbiota, namely Verrucomicrobia and Actinobacteria, are not as clearly elucidated in the context of diet in combination with generational-induced changes. However, Verrucomicrobia (and certain probiotic members of that phylum, i.e., *Akkermansia muciniphila*), a species with anti-inflammatory and immunoregulatory properties^92–97^, emerged as one of the top three drivers of effects of paternal diet on F_1_ offspring community composition (Fig. 5B). Additionally, *A. muciniphila* has been shown to increase glucose homeostasis in diet-induced obesity^96–100^. Predictive random forest modeling demonstrates a clear delineation between members of Firmicutes, such as *Ruminococcus* sp. and *Lactobacillus* sp., and members of the Actinobacterium phylum, such as *Bifidobacterium pseudolongum*, in determining the classification of offspring gut microbiomes. Surprisingly, paternal HFD and associated gut microbial changes had the strongest predictive accuracy in determining offspring classification of the experimental factors analyzed despite more temporally proximal events taking place during the offspring’s lifespan (i.e., maternal care and odor exposure). Maternal investment indicators such as nursing, licking, and grooming early in the offspring life period also are influenced by paternal high fat diet^11^ and thus have strong predictive accuracy.

*Bifidobacterium pseudolongum* was highly expressed in the offspring of HFD sires, specifically those raised in SNH conditions. This species has recently been shown to reduce body weight, visceral fat, and reverse HFD-induced increases in Firmicutes to Bacteroidetes ratios^101^. We hypothesize that *Bifidobacterium pseudolongum* potentially acts as a compensatory response within the gut microbiota to elevated levels of Firmicutes. *Bifidobacterium pseudolongum* is also associated with decreases in *Akkermansia muciniphila,* replicated in offspring reared in SNH, and increased colonic mucus layer thickness^102^. However, the specific subspecies and strain identified here would need to be known for potential probiotic treatment, as *Bifidobacterium pseudolongum* has many strains with varied metabolic effects^103^. Future work will also be required to disentangle the influence that paternal diet, maternal care and rearing environments have on differential expression of these buffering microbiome species.

Recent research has identified several potential routes for non-genetic inheritance of paternal experience, including the seminal microbiome, epigenetic alterations to sperm, paternal inheritance of mitochondrial DNA, and maternal investment. For example, diet-induced alterations to paternal microbiome may be transferred to offspring via the seminal fluid microbiome^28,104^. Others have identified specific alterations in sperm epigenetic mechanisms, including small noncoding RNAs (sncRNAs), tRNAs^22,24,29^, and microRNAs^25,26,105^; these mechanisms alter gene expression of developing embyos^106^ and likely contribute to metabolic insults in offspring^10,25,86^. However, the role of maternal investment should not be overlooked. We have previously demonstrated that female rats have a preference for lean males when given the option between a lean vs. DIO male^11^ and this is reflected in quality of maternal care. Others have identified increased maternal behavior based on the female’s perceived quality of a mate^19–21^. However, the total impact of transgenerational insults has not been explored in the context of mate preference. The influence of maternal preference can be reduced by utilizing in vitro fertilization^9,24,107^ or cross-fostering techniques^105–109^, though both of these techniques can alter development^110,111^. Maternal investment can also be manipulated by the quality of the environment, as an enriched or naturalistic rearing environment will promote a higher proportion of high-quality vs. low-quality maternal care^11,43,51,83^.

### Limitations

While the statistical analyses and data reported here are robust and replicable, we acknowledge that there are some limitations to this study that prevent us from concluding the underlying functional mechanism(s) by which paternal high fat diet, maternal housing, and acute stress (predator odor exposure) influence the gut microbiota throughout the offspring lifespan. Fecal and stomach sampling took place at a single discrete timepoint in F_1_ offspring peri-adolescence^112^; future studies using longitudinal sampling would permit us to determine if factors such as maternal care or rearing condition immediately impact the offspring microbiome or if these changes are the result of gradual interactions between maternal care and rearing condition. Future work should also consider longer-lasting and aging-related changes in offspring as they progress from peri-adolescence to adulthood while considering the possibility that these changes may expand to future generations as well. Moving forward, careful design and implementation of experiments investigating these complex and interacting factors will be required to fully elucidate the influence of paternal experience on maternal investment and rearing environment on offspring metabolism and behavior.

## Conclusions

Preconception and early-life factors have a significant influence on the development and adult behavior of many organisms, including mammals. Here, we show that preconception paternal HFD feeding, early-life rearing environment, and maternal care influence offspring weight, behavior and the diversity of their microbiome. Specifically, we have identified that paternal HFD positively associates with offspring weight and anxiety-like behaviors in peri-adolescence, and maternal investment inversely associates with offspring weight. We show that predator odor exposure is an acute stressor that impacts offspring gut microbiota as assessed by richness and evenness indices of alpha-diversity as well as global compositional changes in beta-diversity measurements. These data suggest that parental conditions such as paternal HFD and maternal care, together with acute stress exposures during early life, could potentially impact the gut microbiota through adulthood. Future studies are required to determine the germline mechanisms driving these generational, and potential transgenerational, effects. However, the role of maternal investment should not be overlooked^21^ when determining priming of offspring development and behavior by paternal experience. Overall, these data support the hypothesis that both paternal diet and maternal care have profound influence on offspring microbiota diversity and community composition, and that these changes influence the behavior of peri-adolescent offspring.

## Supplementary Information

Supplementary Tables.

Supplementary Information.

## Data Availability

Mapping files and raw short-read DNA sequences are publicly available on the microbiome study management platform Qiita (http://qiita.ucsd.edu/study/description/1634) and the European Molecular Biology Laboratory—European Bioinformatics Institute (EMBL-EBI, European Nucleotide Accession No. ERP015380). Other data from the study are available from the corresponding author on reasonable request.

## Abbreviations

16S rRNA: RNA component of the 30S subunit of a prokaryotic ribosome
ABN: Arched-back nursing
ACTH: Adrenocorticotropic hormone
ASV: Amplicon sequence variant
AUC: Area-under-the-curve
CD: Control diet
CLR: Center-log ratio
CO: Control odor
*Crf*: Gene encoding corticotropin-releasing factor
DIO: Diet-induced obesity
EPM: Elevated plus-maze
F_0_: Filial 0/parental
F_1_: Filial 1/offspring
FPR: False-positive rate
GxE: Gene by environment interaction
HFD: High-fat diet
HPA: Hypothalamic–pituitary–adrenal
LG: Licking and grooming
OFT: Open-field test
OTU: Operational taxonomic unit
PCoA: Principal coordinates analysis
PD: Postnatal day
PO: Predator odor
PPT: Partner preference test
PVC: Polyvinyl chloride
ROC: Receiver operating characteristic
rRNA: Ribosomal ribonucleic acid
SH: Standard housing
SNH: Semi-naturalistic housing
TPR: True-positive rate
UniFrac: Unique fraction metric
