# Supplementary material for: Effects of paternal high-fat diet and maternal rearing environment on the gut microbiota and behavior
Source: Sci Rep. 2022 Jun 17;12:10179. doi: 10.1038/s41598-022-14095-z (PMC9205913; doi:10.1038/s41598-022-14095-z)
Supplement: Supplementary file 1 — Supplementary Tables. [file 41598_2022_14095_MOESM1_ESM.docx]

**Tables**

Supplemental Table 1. Stomach microbiome alpha-diversity in samples from F_0_ males.

| Alpha-Diversity | Group 1 | Group 2 | H | *p*-value | Kruskal-Wallis Factor |
| --- | --- | --- | --- | --- | --- |
| Shannon’s H’ | CD (*n* = 6) | HFD (*n* = 5) | 4.80 | 0.028 | **Diet** |
|  | High (*n* = 5) | Low (*n* = 6) | 4.80 | 0.028 | **Rearing in the open field arena** |
| Simpson’s evenness | High (*n* = 6) | Low (*n* = 5) | 4.80 | 0.028 | **Line crosses in the open field arena** |

Supplemental Table 2. Stomach microbiome alpha-diversity in samples from F_1_ offspring.

| Alpha-Diversity | Group 1 | Group 2 | H | *p*-value | Kruskal-Wallis Factor |
| --- | --- | --- | --- | --- | --- |
| Chao1 | SH (n = 9) | SNH (n = 14) | 4.86 | 0.027 | **Housing condition** |
|  | High (n = 11) | Low (n = 11) | 6.06 | 0.014 | **Rearing in open field arena** |
| Menhinick | High (n = 11) | Low (n = 11) | 5.14 | 0.023 | **Rearing in open field arena** |
| Simpson’s evenness | SH (n = 9) | SNH (n = 14) | 7.34 | 0.007 | **Housing condition** |
|  | Female (n = 14) | Male (n = 9) | 5.14 | 0.023 | **Sex** |
|  | High (n = 12) | Low (n = 10) | 4.18 | 0.041 | **Rearing in plus maze** |
|  | CO (n = 9) | PO (n = 14) | 5.14 | 0.023 | **Odor exposure** |

Supplemental Table 3. Alpha-diversity of fecal microbiomes in samples from F_1_ offspring.

| Alpha-Diversity | Group 1 | Group 2 | H | *p*-value | Kruskal-Wallis Factor |
| --- | --- | --- | --- | --- | --- |
| Faith's PD | CD/SH/PO (*n* = 6) | CD/SH/CO (*n* = 4) | 6.55 | 0.011 | **Manipulation** |
|  | CD/SH/CO (*n* = 4) | HFD/SH/CO (*n* = 6) | 5.50 | 0.019 | Overall p-value: 0.018 |
|  | HFD/SH/CO (*n* = 6) | HFD/SNH/CO (*n* = 5) | 5.63 | 0.018 | Overall H-statistic: 16.952 |
|  | High (*n* = 21) | Low (*n* = 20) | 4.35 | 0.037 | **Rearing in plus maze** |
| Observed OTUs | CD/SH/PO (*n* = 6) | CD/SNH/PO (*n* = 6) | 4.35 | 0.037 | **Manipulation** |
|  |  | CD/SH/CO (*n* = 4) | 6.59 | 0.010 | Overall p-value: 0.021 |
|  | CD/SH/CO (*n* = 4) | HFD/SH/CO (*n* = 6) | 5.50 | 0.019 | Overall H-statistic: 16.537 |
